# Shade Avoidance Restricts Soybean Breeding Progress and Increases Herbivore Susceptibility

**DOI:** 10.1111/eva.70280

**Published:** 2026-06-12

**Authors:** Lukas Vonmetz, Emanuel B. Kopp, Simon Jäggi, Pascal A. Niklaus, Samuel E. Wuest

**Affiliations:** ^1^ Group Breeding Research, Division Plant Breeding Agroscope Waedenswil Switzerland; ^2^ Department of Evolutionary Biology and Environmental Studies University of Zurich Zurich Switzerland; ^3^ Zurich‐Basel Plant Science Center Zurich Switzerland

## Abstract

High planting densities expose field crops to competition for light, which typically induces shade avoidance responses such as stem elongation. While adaptive in natural environments, these responses can lower yield and increase susceptibility to stress in agricultural systems. We tested whether soybean breeding over the past century has altered shade avoidance and associated trade‐offs. Twenty‐one Canadian cultivars released between 1922 and 2018 were grown in pots under either control or shade‐avoidance‐inducing light conditions, achieved by altering reflected light spectrum without reducing photosynthetic radiation. Plants exposed to shade‐avoidance‐inducing light grew taller and suffered greater thrips damage, consistent with expectations of increased stem elongation and reduced defence. More recent cultivars showed higher susceptibility to thrips than older ones. Breeding progress in seed yield was driven largely by greater biomass allocation to seeds and reduced branching. However, under shade‐inducing light, the yield improvements were smaller, pointing to shade avoidance as a limiting factor. Our results indicate that while soybean breeding has improved yield and shifted morphology towards ideotypes suited for high‐density stands, persistent shade avoidance responses constrain breeding progress for yield and increase herbivore susceptibility. Breeding strategies that reduce sensitivity to neighbor cues may therefore improve soybean productivity and resilience.

## 1 Introduction

Densely populated environments impose strong selection on plant traits that improve access to limiting resources, particularly light. Shade avoidance responses thus evolved as an adaptive strategy to avoid shade and more efficiently compete for light (Schmitt 1997; Schmitt et al. 2003); this includes stem and petiole elongation, leaf angle adjustment, reduced branching or shifts in phenology (Ballaré et al. 1990; Franklin 2008; Pierik and De Wit 2014; De Wit et al. 2016; Gruntman et al. 2017). These responses are primarily triggered by a reduction in the ratio of red to far‐red (R:FR) light, which signals the presence of neighbouring vegetation (Ballaré et al. 1990). While advantageous for individual fitness in competitive environments, these responses often involve resource allocation trade‐offs and can result in decreased allocation to storage and reproduction or increased susceptibility to lodging (Schmitt et al. 1995; Franklin and Whitelam 2005).

In agricultural systems, plants are typically grown in uniform stands where overall yield, rather than individual performance, determines success (Duvick et al. 2004). In this context, shade avoidance responses—while adaptive in nature—can reduce yield by reallocating resources from harvestable organs toward elongation or support (Boccalandro et al. 2003; Carriedo et al. 2016). This notion is a core concept of ‘Darwinian Agriculture’ (also known as ‘Evolutionary Agroecology’), and suggests that traits which evolved to enhance individual competitiveness may conflict with collective crop performance (Weiner 2004, 2019; Denison 2012). This view challenges the assumption that breeding progress leads to an ‘improvement’ of crops and posits that evolutionary trade‐offs persist in modern breeding programs and may lead to conflicting selective responses, depending on the breeding scheme chosen.

Breeding of field crops typically involves a phase early in the breeding cycle predominantly characterized by individual‐level selection (e.g., during mass selection), followed by a phase of predominant selection at the level of groups of highly related individuals (e.g., rows or plots of inbred progenies, leading to multi‐level selective regimes; Murphy et al. 2017, Fischer 2020). This may lead to conflicting selection pressures on ‘selfish’ traits: early individual‐level selection favours selfish traits, as competitive individuals stand out against their less competitive neighbours and increase in frequency. Conversely, later group selection favours less competitive traits. Despite this conflict, the analysis of historic series of cultivars released over several decades has revealed that with breeding for higher yields, other traits that are specifically beneficial for densely sown plant populations also increased: vertical leaf angles, steeper roots, higher water and nitrogen use efficiency, etc., (Costa Netto et al. 2025; Duvick et al. 2004; York et al. 2015; Zhu et al. 2019, 2022). At the same time, breeders have also developed idealized plant models, originally denoted as “communal” ideotypes, that express such traits benefiting the group (Donald 1968). A well‐known example is the reduction in plant height in wheat (Reitz and Salmon 1968) and rice (Jennings 1964), and recently maize, which increases yield potential under high densities (Kosola et al. 2023). Together, these phenotypic changes therefore reflect deliberate or inadvertent shifts away from competitive traits and towards traits that support collective performance (Denison 2012; Weiner 2019). However, current breeding practises do not guarantee that traits that benefit the group at the expense of individual fitness will automatically be favoured during selection.

From this perspective, limiting shade avoidance as a selfish response to neighbour plants could improve stand‐level yield (Golan et al. 2023; Liu et al. 2020; Li et al. 2023, 2024). Shade avoidance is a plastic trait, however, and compared to other, often more fixed, traits, relatively less is known about how it has changed with crop breeding progress (Wille et al. 2017). In wheat, genetic changes that reduce plant height indirectly also reduce shade avoidance responses (Colombo et al. 2022; Golan et al. 2024). While there is evidence for genetic variation in shade avoidance response in soybean (Gong et al. 2015), it remains unclear whether the responses of modern soybean cultivars differ from those of historical cultivars.

Studying shade avoidance responses is complicated by the fact that plants can respond to different aspects of the light environment, such as changes in light levels and changes in light spectrum (Ballaré and Pierik 2017). Changes in the light environment of a plant facing a competitor is also typically confounded with various types of resource competition, such as belowground competition for soil nutrients, which also influences plant growth (Murphy and Dudley 2007; Kopp et al. 2025). To manipulate the spectral properties of light, filters are often placed above plants (e.g., Golan et al. 2023), but these typically also attenuate light intensity, making it harder to isolate the specific role of light level and light spectrum.

Here, we studied the effect of breeding in soybean on shade avoidance responses using 21 cultivars released between 1922 and 2018. We positioned light filters beneath plants to manipulate the spectrum of reflected light, specifically the red to far‐red ratio and amounts of blue light. Because we did not manipulate incident light, levels of radiation available for photosynthesis remained largely unchanged (Green‐Tracewicz et al. 2011). Since we grew single plants in pots, the effect of light quality changes also was not conflated with effects of belowground competition. Specifically, we were interested in whether modern soybean cultivars exhibit reduced shade avoidance, which is likely an inadvertent adaptation to higher planting densities and improved group‐level yield.

## 2 Materials and Methods

### 2.1 Shade Avoidance Experiment

**FIGURE 1:**
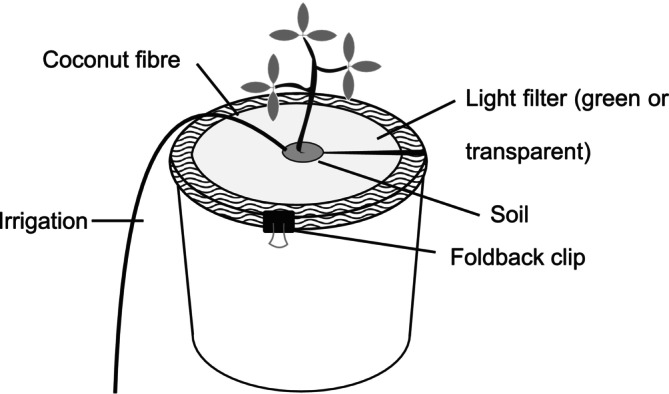
Schematic representation of the experimental setup. Each plant was cultivated in an individual pot, the soil surface of which was covered with a coconut fibre disc. A light filter was affixed directly to the upper surface of the disc. To ensure mechanical stability during field experiments, the disc–filter assembly was secured using foldback clips. The filter is either transparent, not altering the light wavelength composition; or green, reducing the amount of red and blue light reflected. Plants sense the presence of neighbours through shifts in light spectrum and might adapt their growth strategy accordingly (Figure S1).

In summer 2024, a pot experiment was performed in Wädenswil, Switzerland (N 47°13′19.7″ E 8°40′8.4″) to investigate shade avoidance responses in a set of 21 early‐maturing soybean cultivars released in Canada between 1922 and 2018 (Table S1). Seeds were germinated in small pots (0.29 L, filled with FloraDur A‐Block, Floragard Vertriebs‐GmbH, Germany) in the glasshouse (night: 16°–18°C, day: 22°C–24°C, photoperiod: 12 h). Pots were placed on four large tables, with a distance of 18 cm between pots. The pots were covered by a large plastic sheet with holes allowing plants to grow through. Two tables were covered with a clear plastic sheet not altering the spectrum of the reflected light (control treatment; Clear 130, LEE Filters, UK). The other two tables were covered with a green plastic sheet that particularly reduced the red to far red ratio of reflected light (shade‐avoidance inducing treatment, SAI; Fern Green 122, LEE Filters, UK); as used in previous studies (Chen et al. 2023; Wille et al. 2017; Gruntman et al. 2017; Golan et al. 2024). After 10 days, 12 plants per cultivar and treatment were transplanted into 3 L pots (21 cultivars × 2 treatments × 12 replicates = 504 pots) filled with a special mixture of soybean soil (RICOTER Erdaufbereitung AG, Frauenfeld, Switzerland: 25% garden soil max. 15 mm; 60% white peat 0–30 mm; 15% perlite 2–6 mm). Each pot's upper surface was covered at soil level by a round piece of plastic sheet (green or transparent according to the treatment, Figure 1 and Figure S1). A coconut fibre disk was inserted between the plastic sheet and the soil to reduce warming and to improve air circulation and evaporation. Measuring the reflectance spectra of these setups (FieldSpec‐3 spectroradiometer, ASC Inc. Boulder, Colorado; Figure S2) confirmed that the green filter setup effectively reduced reflected light in the UV/blue range, and strongly in the red range, resulting in a corresponding reduction of the red/far red ratio. The pots with the two treatments were randomly arranged in a grid pattern in a horticultural tunnel covered by a hail protection net, with a minimum distance of 46 cm between pots to diminish light competition between plants. Plants were irrigated ad libitum. For each cultivar and treatment, four plants were randomly selected and harvested at physiological stages R2 (full bloom), R6 (full seed stage, characterized by green pods containing fully developed green seeds), and R8 (full maturity, marked by leaf abscission and pods attaining their mature colour; Fehr and Caviness 1977). Plant roots were washed, and shoots and roots dried (40°C, min 48 h) and weighed. At full maturity, soybean pods were threshed and plant level seed yield determined. No plants of the cultivar ‘Capital’ were collected at R2 due to insufficient planting material resulting from poor germination (Table S2). However, this did not affect any of the main analyses and results of the paper, the focus of whose was on later developmental stages, including final harvest. During the early growth period, leaf damage caused by thrips was observed. Therefore, leaf damage was scored visually 36 days after sowing (09.07.2024), using an ordinal scale ranging from 0 (no damage); 1 (few punctures on 1–2 leaves); 2 (punctures on more than two leaves) or to 3 (punctures on multiple leaves, incl. small holes and slight deformations of leaves), see Figure S3 for representative examples. Afterwards, all plants were treated with insect‐repellent and no additional thrips or thrips damages were seen.

### 2.2 Statistical Analysis

All statistical analyses were performed in R version 4.4.3 (www.r‐project.org). Multilevel linear models were fitted using the aov‐function using its Error() option (version 4.5.0). Fixed effects included year of release (YOR, continuous variable) and SAI treatment (categorical: shade‐avoidance inducing or control), and the interaction of both terms. Error terms were cultivar, and the interaction of genotype with the SAI treatment. Effects of YOR therefore were tested using cultivar as error stratum, and the YOR × SAI treatment interaction used cultivar × SAI as error term. All dependent variables were natural log‐transformed to meet model assumptions (especially homoscedasticity) and to render comparisons between individuals of different sizes more biologically meaningful.

For the analysis of the herbivore damage, a cumulative‐link mixed model was fitted using the *clmm2()* function from the *ordinal* package. Thrips damage was treated as an ordinal response, with the fixed and error terms as described above for the linear models.

## 3 Results

### 3.1 Plants Under SAI Treatment Suffered From Greater Herbivore Damage at Early Growth Stage

**FIGURE 2:**
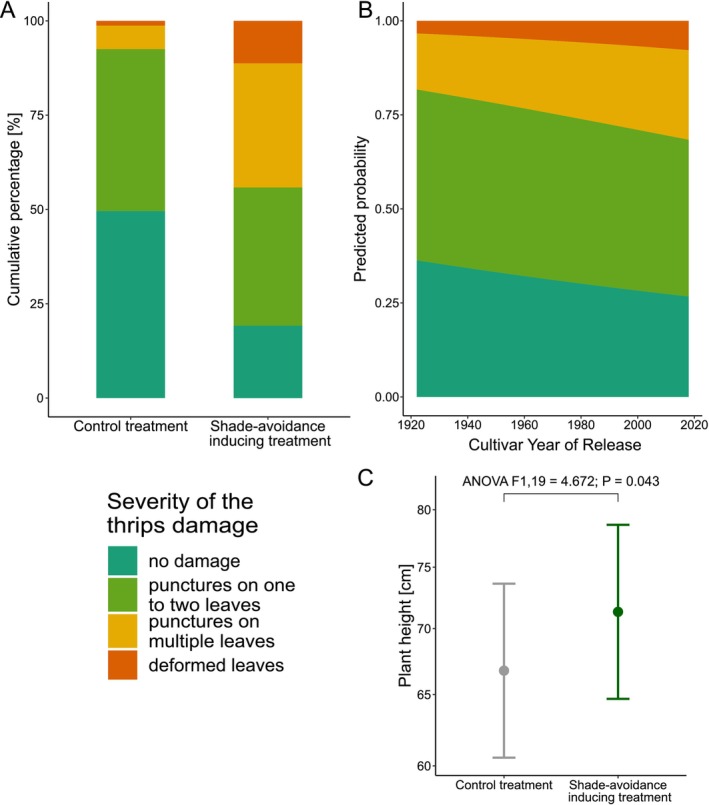
(A) Comparison of the thrips damage at growth stage V4 between the two treatments as cumulative percentage of the four severity categories. Plants under the shade‐avoidance inducing treatment suffer higher thrips damages than control plants. (B) Predicted probability of infestation over the cultivar year of release. More modern cultivars show higher thrips damages than historic cultivars. (C) Although the plants in the shade‐avoidance inducing treatment at growth stage V4 showed higher susceptibility for thrips damages, at full height (growth stage R6) they were significantly taller compared to the control treatment, suggesting a shade‐avoidance elongation response. The points and error bars represent the estimated marginal means from the mixed effects model ±SE. Plant height is depicted on the logarithmic scale.

After 36 days of growth (growth stage V4), we observed the presence of thrips in our experiment, often accompanied by early signs of leaf puncturing. Before treating against these unexpected pests, we visually quantified feeding signs on each plant individual. Plants growing under SAI treatment showed significantly more damage from thrips than plants under the control ratio treatment (odds ratio = 0.356, *p* = 0.001; Figure 2A), in line with known trade‐offs between defence against herbivores and competition for light (Cipollini 2004; McGuire and Agrawal 2005). Also, modern cultivars were more susceptible to thrips than historical cultivars (odds ratio = 1.006, *p* = 0.025; Figure 2B). After treating plants against thrips, no damage was observed on new leaves until the end of the experiment.

At full height (growth stage R6), plants were taller in the SAI than in the control treatment (ANOVA *F*_1,19_ = 4.672, *p* = 0.043; Figure 2C and Figure S4a), in accordance with known shade avoidance responses. This suggests that the effects observed in the later growth stages are indeed due to shade avoidance responses and not to result of earlier leaf damage by thrips, since the latter would have reduced growth. However, we did not measure specific physiological changes (e.g., cell/internode elongation etc.) that could have affected final plant height. Finally, the shade‐avoidance inducing treatment also reduced root biomass, and the number of branches counted at harvest stage (R8, Figure S4b), as expected from previous reports (Green‐Tracewicz et al. 2011).

### 3.2 Modern Cultivars Are More Productive and Differ in Shoot Architecture

**FIGURE 3:**
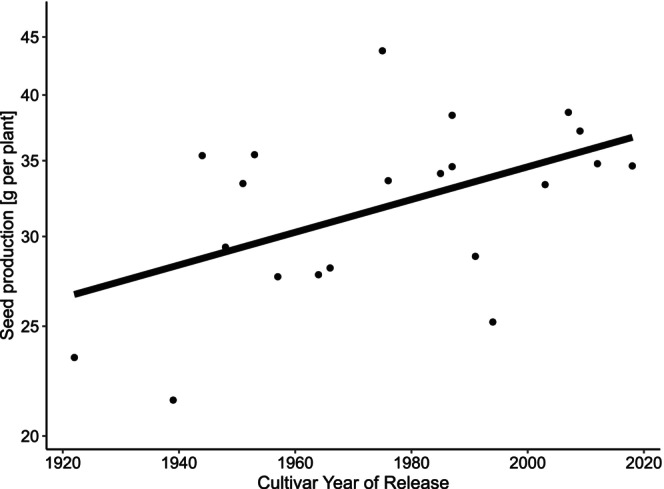
Cultivars released from 1922 to 2018 showed a significant increase in seed productivity at the individual plant level (21 genotypes; ANOVA *F*_1,19_ = 6.594, *p* = 0.018). The points correspond to the mean seed production (depicted on a logarithmic scale, *n* = 8 for each cultivar) of the 21 soybean cultivars used in the experiment.

Seed production significantly increased with year of release of the cultivars (ANOVA *F*_1,19_ = 6.594, *p* = 0.018), indicating breeding progress (Figure 3). At the same time, the number of branches per plant decreased with year of release (ANOVA *F*_1,19_ = 10.97, *p* = 0.003). Hence, modern cultivars did not produce more seeds by producing a greater number of equally productive branches, but by increasing the productivity per branch. Additionally, no change in stover production over time was observed, indicating that vegetative biomass (shoot biomass excluding yield) did not increase through breeding (Table S3). However, modern cultivars tended to be more compact than historical cultivars, shown by an increasing ratio of shoot biomass to plant height over the last century (ANOVA *F*_1,19_ = 3.3, *p* = 0.085). This compactness may contribute to improved plant stability or resource use efficiency. Thus, breeding successfully led to an increase in plant productivity over time, while simultaneously changing the cultivars' architecture.

**FIGURE 4:**
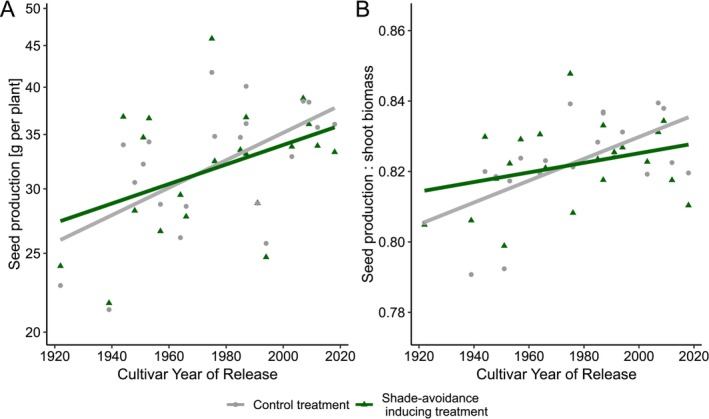
(A) The breeding progress was higher in the control treatment than in the shade‐avoidance inducing treatment, as shown by the steeper slope of the grey line compared to the green one (ANOVA *F*_1,19_ = 4.771, *p* = 0.041). The grey dots correspond to the mean seed production of a cultivar under the control treatment (*n* = 4), whereas the green triangles are the mean seed production per cultivar under the SAI treatment (*n* = 4). Values depicted on a logarithmic scale. (B) The control treatment shows a steeper increase towards a more favourable seed production to shoot biomass—ratio (ANOVA *F*_1,19_ = 6.339, *p* = 0.021).

Finally, breeding progress of seed production was higher in control than under the SAI treatment (interaction of YOR × treatment on seed production; ANOVA *F*_1,19_ = 4.771, *p* = 0.041; Figure 4A). This progress was, however, not due to increased total biomass, but to a large degree due to an increased allocation of biomass to seeds. Indeed, the change of allocation over breeding history was lower under the SAI treatment (interaction of YOR × treatment on seed production—to—shoot biomass ratio; ANOVA *F*_1,19_ = 6.339, *p* = 0.021; Figure 4B).

## 4 Discussion

We investigated the effects of altered light conditions on growth and biomass allocation in soybean cultivars released over nine decades of Canadian breeding history. Plants exposed to simulated light competition were more susceptible to thrips damage than control plants, consistent with expectations due to shade avoidance responses (McGuire and Agrawal 2005; Ballaré and Pierik 2017). Moreover, more recently released cultivars exhibited higher thrips damage than older ones. At the same time, we observed clear breeding progress for seed production, with modern cultivars producing more seeds than historical cultivars. However, this increase with year of release was steeper under control conditions than under SAI treatment mimicking the presence of neighbours. The gain in seed production was largely driven by increased biomass allocation to seeds, but this trend weakened under shade avoidance conditions. Taken together, these findings indicate a combination of both positive, that is, intended, but also inadvertent responses to breeding. In the following, we interpret these patterns in light of evolutionary trade‐offs, the challenge of selecting for plastic versus fixed traits, and the possibility that breeding schemes do not fully resolve conflicts between individual fitness and group‐level performance (the ‘Darwinian Agriculture’ perspective; Donald 1968; Zhang et al. 1999; Denison 2012; Weiner et al. 2017, Weiner 2019).

### 4.1 Modern Cultivars Are Both More Productive and Closer to the Soybean Ideotype

Breeding has improved traits beneficial to crop production, including allocation to seeds and stand performance. Soybean yield has greatly increased in the last century, and the genetic gain through breeding progress has been documented in multiple studies (Luedders 1977; Specht et al. 1999; Cober et al. 2005; Kahlon et al. 2011). Although yield is typically evaluated at the stand level, this trend was also evident at the individual plant level in our experiment, where modern cultivars allocated more biomass to seeds. In addition, modern cultivars show morphological changes in agreement with a soybean ideotype well‐suited for dense plantings, the latter of which includes fewer branches, shorter internodes, and erect and narrow leaves (Kokubun 1988; Bianchi et al. 2020; Lyu et al. 2023).

Importantly, these traits, and the increased allocation to seeds, are relatively stable across environments, in contrast to more plastic responses such as shade avoidance, which is triggered by changes in light conditions. Distinguishing such ‘fixed traits’ that are readily included in an ideotype from environmentally induced plastic responses may help explain why some breeding outcomes enhance yield, whereas others constrain it under competition (Weiner 2004). Future breeding efforts may therefore benefit from incorporating ‘plastic ideotypes’ and define trait responses that are desirable or unwanted for improved crop performance.

### 4.2 Stronger Shade Avoidance and Increased Herbivore Susceptibility in Modern Cultivars

In contrast to above‐mentioned improvements, our data highlight breeding‐associated changes likely to be undesirable. First, modern cultivars exhibited signs of a stronger response to the shade‐avoidance inducing treatment than older cultivars, including a stronger reduction in resource allocation to seeds. While some shade avoidance responses may support canopy optimization or increase yield stability under variable emergence rates (Zhou et al. 2024; Pereyra et al. 2026), their persistence could also reflect selection for traits that enhance individual competitiveness during early breeding stages (discussed below).

Multiple studies have shown trade‐offs between competition and herbivore susceptibility (McGuire and Agrawal 2005; Pellissier et al. 2018), a special case of the wider growth‐defence trade‐off (Herms and Mattson 1992; Fiorucci 2020). Light signals, particularly red‐to‐far red ratios perceived by plant phytochromes, influence jasmonate and auxin hormonal pathways that likely regulate this balance. In particular, low red: far‐red ratios lead to phytochrome B inactivation, which can suppress jasmonic acid signalling and reduce herbivore defences, for example through the accumulation of JAZ repressors that inhibit JA‐responsive pathways. At the same time, inactive phytochrome B promotes PIF‐mediated expression of auxin biosynthesis genes associated with shade avoidance responses and stem elongation (Moreno et al. 2009; Pierik and Ballaré 2021). Together, these molecular responses provide a mechanistic explanation for reduced herbivore resistance when plants prioritize competitive growth over defence investment. Consistent with this view, plants exposed to SAI treatment in our experiment showed increased thrips damage. Although we did not directly measure defence traits, this aligns with evidence that shade avoidance reduces investment in defence pathways (Cerrudo et al. 2012, 2017; Ballaré 2014). We cannot fully exclude direct effects of light treatments on thrips behaviour; however, this appears unlikely given that thrips are primarily attracted to blue rather than green wavelengths (Stukenberg et al. 2020). Instead, many of the phenotypic changes observed are consistent with a shade avoidance response (increase in plant height, reduced root allocation, and reduced branching etc.), and that this supports a plant‐mediated mechanism that led to increased herbivore damage. In crops, especially in monocultures, such responses may be disadvantageous for two reasons. First, uniform elongation in response to competition cues may reduce yield through suboptimal resource allocation. Second, reduced defence can increase pest damage, leading to indirect yield losses (Pierik and Ballaré 2021).

### 4.3 Evolutionary Constraints May Persist in Modern Soybean Breeding

Breeding for yield in many field crops has historically been associated with improved adaptation to high planting densities, often through the inadvertent selection of traits that reduce competition among neighboring plants (Zhang et al. 1999; Denison 2012; York et al. 2015; Weiner et al. 2017; Cossani and Sadras 2021). In maize, for example, traits such as erect leaves, reduced tassel size, and altered root architecture enhance resource use efficiency and reduce interference (e.g., Duvick et al. 2004; York et al. 2015) and can also be interpreted as cooperative adaptations that improve group‐level performance.

In soybean, evidence for improved tolerance to higher planting densities in modern varieties remains relatively limited (Cober et al. 2005; De Bruin and Pedersen 2009; Suhre et al. 2014; Carciochi et al. 2019). One explanation is that traits enhancing individual competitiveness have been retained during selection and continue to limit yield improvement at higher densities. Consistent with this, we observed reduced breeding gains in terms of allocation to seeds under SAI conditions relative to controls.

It is, of course, possible that the plastic responses we observe under SAI treatment may also have beneficial impact on crop performance, for example by optimizing canopy structure or plants' architecture. However, the association of these plastic responses with increased herbivore susceptibility represents a clear trade‐off. Moreover, experimental studies have shown that reduced height and attenuated shade avoidance can substantially increase yield under dense planting, particularly at northern latitudes (Lyu et al. 2023; Li et al. 2023, 2024; Qin et al. 2023). Selection schemes typically contain both elements of individual‐level selection (e.g., within rows of plant individuals with low to moderate relatedness) and group‐level selection (e.g., amongst plots; with high within‐plot relatedness; see Murphy et al. 2017; Fischer 2020; Biernaskie 2022; for details). Strong selection in early generations, often among competing individuals, is likely to favour traits that enhance competitiveness. This may lead to the retention of shade avoidance responses, contributing to suboptimal performance under dense cropping conditions (Keuskamp et al. 2010; Bongers et al. 2018; Chen et al. 2023).

Several strategies could help to mitigate this limitation. First, breeding programmes could place greater emphasis on group‐level selection, for example by shifting resources to selection on plot‐level performance (Biernaskie 2022). The genomic selection approach will facilitate this by enabling model training and subsequent prediction based on group‐level performance (Crossa et al. 2017). Second, breeding efforts could focus on the development of ‘plastic ideotypes’ that explicitly account for environmentally induced responses and aim to attenuate less desired plasticity such as excessive shade avoidance. Third, selection schemes could be modified to reduce the intensity of early‐generation competition, for example by increasing relatedness among neighbouring plants, lowering planting densities or minimising competitive interactions during early selection stages. For example, in the soybean breeding programme in Novi Sad, Serbia, such an approach has been implemented successfully using a single‐pod descent method instead of traditional bulk or pedigree selection (Miladinović et al. 2011).

## 5 Conclusion

While traditional breeding has successfully improved soybean yield and ideotype traits, our results indicate that shade avoidance responses have persisted or even increased in modern cultivars. Potentially favoured because they enhance individual competitiveness during early breeding stages, these responses may constrain crop performance and increase herbivore susceptibility, particularly in dense stands. To fully unlock soybean's yield potential, future breeding efforts should prioritise traits that enhance group‐level performance under dense sowing conditions. This includes reducing potentially disadvantageous plastic responses to light competition and developing ‘plastic ideotypes’ that integrate adaptive environmental responsiveness. Such strategies could lead to crops better optimized for modern agricultural systems, improving both productivity and resilience.

## Funding

This work was supported by Schweizerischer Nationalfonds zur Förderung der Wissenschaftlichen Forschung (310030_192537).

## Conflicts of Interest

The authors declare no conflicts of interest.

## Supporting information


**Figure S1:** Experimental setup of the two treatments.
**Figure S2:** Reflectance spectra of shade‐avoidance inducing (green filters) and control treatment (transparent filters) setups, as utilized in the field (filters on coconut fibre mats, placed underneath plants in pots). Segments indicate absorbance maxima of phytochromes in different states (Pr: red‐induced phytochrome state; Pfr: farred‐induced phytochrome state, Sager et al. 1988).
**Figure S3:** Representative examples of thrips damage scoring scheme (0–3). 0: no damage (not shown); 1: punctures (black arrows) on one to two leaves; 2: punctures on multiples leaves; 3: punctures on multiple leaves, small holes and deformations on leaves (blue arrows).
**Figure S4:** Branch number at stage R8 (A, harvest time) and plant height at stage R6 (B, maximal height stage).
**Table S1:** Description of cultivars utilized in this study.
**Table S2:** Harvest dates for the different growing stages and cultivars.
**Table S3:** Estimates and *p*‐values resulting from the linear mixed models.

## Data Availability

The dataset used in this study is available from the Zenodo data repository (https://doi.org/10.5281/zenodo.17183257).
