# Supplementary material for: Shade Avoidance Restricts Soybean Breeding Progress and Increases Herbivore Susceptibility
Source: Evol Appl. 2026 Jun 12;19(6):e70280. doi: 10.1111/eva.70280 (PMC13261778; doi:10.1111/eva.70280)
Supplement: Supplementary file 1 — Figure S1: Experimental setup of the two treatments. Figure S2: Reflectance spectra of shade‐avoidance inducing (green filters) and control treatment (transparent filters) setups, as utilized in the field (filters on coconut fibre mats, placed underneath plants in pots). Segments indicate absorbance maxima of phytochromes in different states (Pr: red‐induced phytochrome state; Pfr: farred‐induced phytochrome state, Sager et al. 1988). Figure S3: Representative examples of thrips damage scoring scheme (0–3). 0: no damage (not shown); 1: punctures (black arrows) on one to two leaves; 2: punctures on multiples leaves; 3: punctures on multiple leaves, small holes and deformations on leaves (blue arrows). Figure S4: Branch number at stage R8 (A, harvest time) and plant height at stage R6 (B, maximal height stage). Table S1: Description of cultivars utilized in this study. Table S2: Harvest dates for the different growing stages and cultivars. Table S3: Estimates and p‐values resulting from the linear mixed models. [file EVA-19-e70280-s001.docx]

## Supplementary material for “Shade avoidance restricts soybean breeding progress and increases herbivore susceptibility”


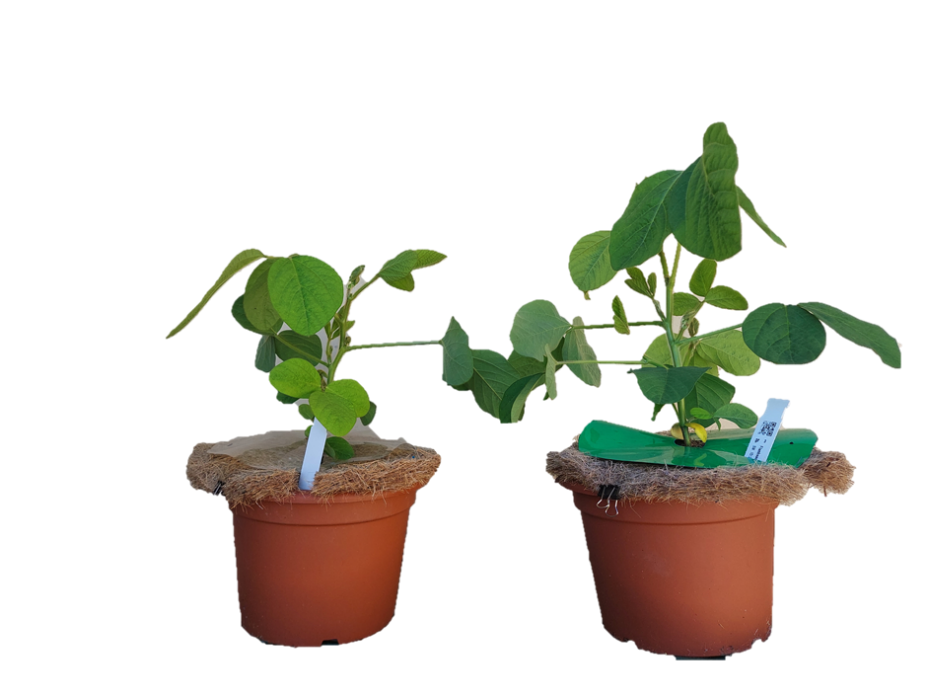
Plant A is growing under the control treatment with a transparent filter above the coconut fibre disc. Plant B is growing under the SAI treatment with a green filter fixed on the coconut fibre disc.

A

B

**Figure S 1: Experimental setup of the two treatments**


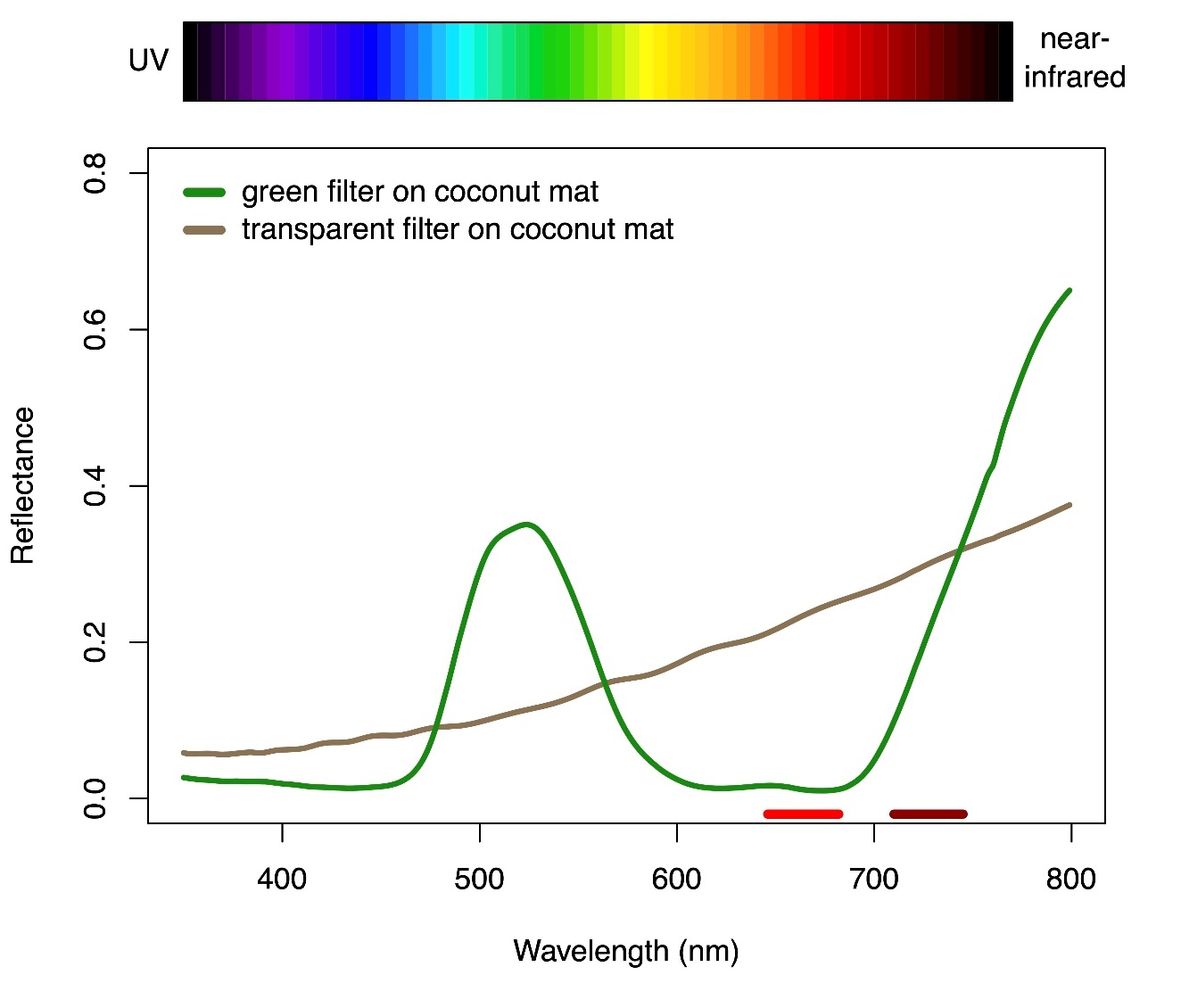


**Figure S2: Reflectance spectra of shade-avoidance inducing (green filters) and control treatment (transparent filters) setups, as utilized in the field (filters on coconut fibre mats, placed underneath plants in pots).** Segments indicate absorbance maxima of phytochromes in different states (Pr: red-induced phytochrome state; Pfr: farred-induced phytochrome state, Sager et al 1988).


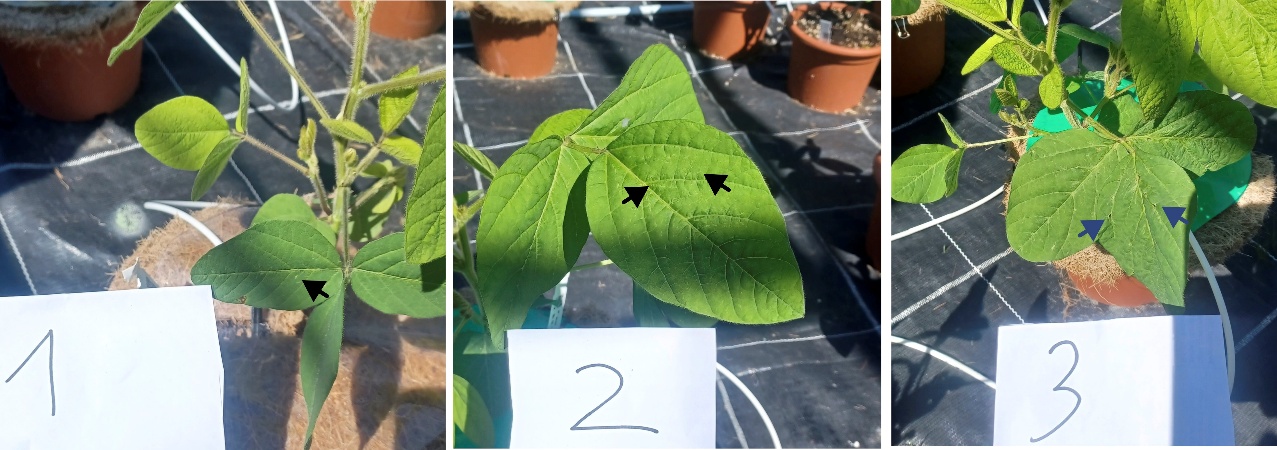


**Figure S3**. **Representative examples of thrips damage scoring scheme (0-3).** 0: no damage (not shown); 1: punctures (black arrows) on one to two leaves; 2: punctures on multiples leaves; 3: punctures on multiple leaves, small holes and deformations on leaves (blue arrows)


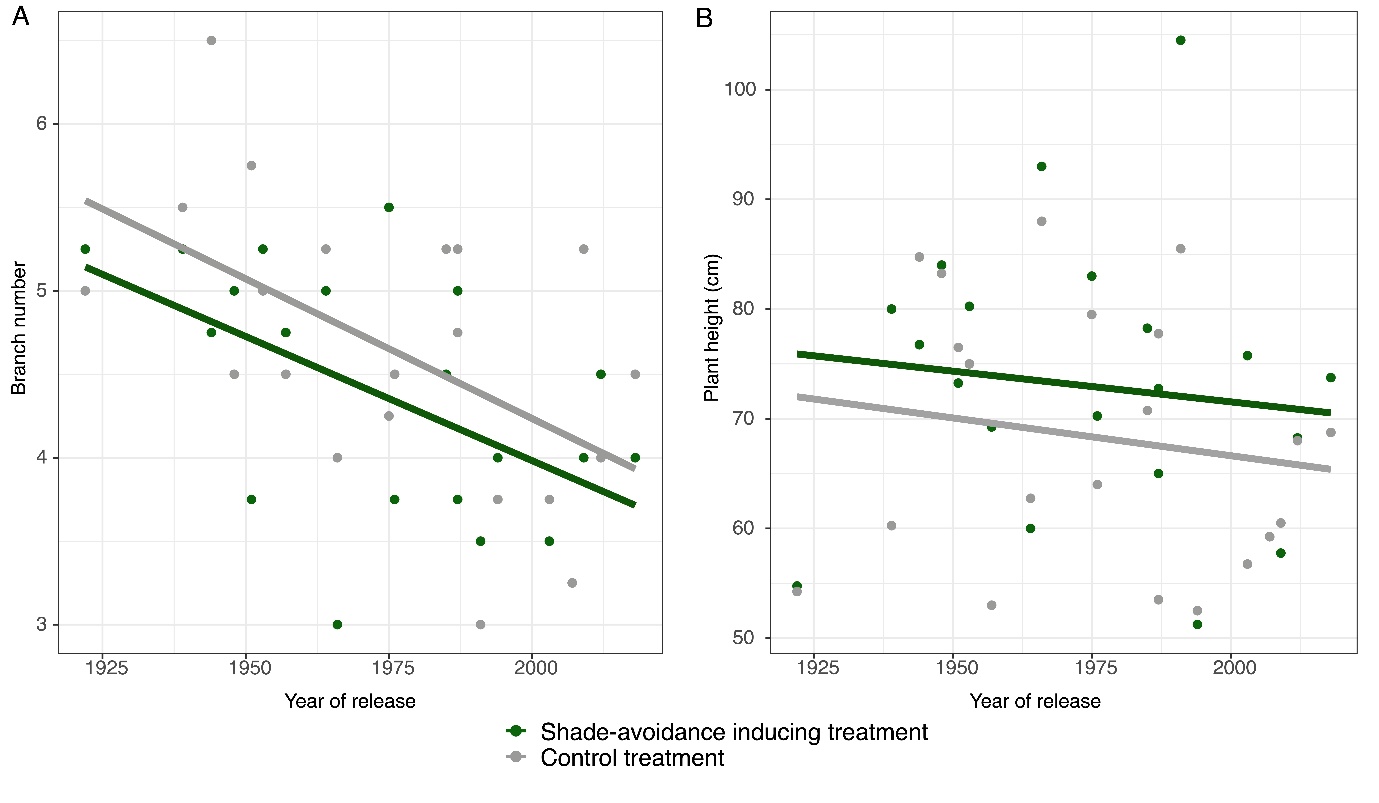


**Figure S4.** **Branch number at stage R8 (A, harvest time) and plant height at stage R6 (B, maximal height stage).**

**Table S 1. Description of cultivars utilized in this study**

We used 21 early maturing cultivars from the Canadian breeding program, spanning almost a century of breeding. NA: not available

| Cultivar | Cultivar year of release | Maturity groups (MG) | Plant introduction number (USDA germplasm system) |
| --- | --- | --- | --- |
| Manitoba Brown | 1922 | OO | PI 548382 |
| Pagoda | 1939 | OO | PI 548398 |
| Capital | 1944 | O | PI 548311 |
| Flambeau | 1948 | OO | PI 548325 |
| Harosoy | 1951 | II | PI 548573 |
| Comet | 1953 | O | PI 548539 |
| Crest | 1957 | OO | PI 548544 |
| Portage | 1964 | OO | PI 548607 |
| Altona | 1966 | OO | PI 548504 |
| Harcor | 1975 | II | PI 548570 |
| Maple Arrow | 1976 | OO | PI 548593 |
| OAC Libra | 1985 | O | PI 548638 |
| Maple Glen | 1987 | OO | PI 548643 |
| OAC Musca | 1987 | O | PI 548644 |
| OAC Vision | 1991 | O | PI 567787 |
| AC Albatros | 1994 | OO | NA |
| OAC Wallace | 2003 | O | NA |
| Naya | 2007 | O | NA |
| Saska | 2009 | O | NA |
| Narita | 2012 | O | NA |
| Ezra | 2018 | OO | NA |

**Table S 2**. **Harvest dates for the different growing stages and cultivars.** We harvested all cultivars at the same phenological states, explaining the difference in harvest dates.

| Cultivar | Harvest VC | Harvest R2 | Harvest R6 | Harvest R8 |
| --- | --- | --- | --- | --- |
| Manitoba Brown | 12.06.2024 | 21.07.2024 | 15.08.2024 | 11.09.2024 |
| Pagoda | 12.06.2024 | 21.07.2024 | 28.08.2024 | 11.09.2024 |
| Capital | 12.06.2024 |  | 02.09.2024 | 11.09.2024 |
| Flambeau | 12.06.2024 | 21.07.2024 | 28.08.2024 | 29.09.2024 |
| Harosoy | 12.06.2024 | 21.07.2024 | 02.09.2024 | 29.09.2024 |
| Comet | 12.06.2024 | 21.07.2024 | 02.09.2024 | 29.09.2024 |
| Crest | 12.06.2024 | 21.07.2024 | 02.09.2024 | 11.09.2024 |
| Portage | 12.06.2024 | 21.07.2024 | 28.08.2024 | 11.09.2024 |
| Altona | 12.06.2024 | 21.07.2024 | 28.08.2024 | 11.09.2024 |
| Harcor | 12.06.2024 | 21.07.2024 | 02.09.2024 | 29.09.2024 |
| Maple Arrow | 12.06.2024 | 21.07.2024 | 02.09.2024 | 29.09.2024 |
| OAC Libra | 12.06.2024 | 21.07.2024 | 02.09.2024 | 29.09.2024 |
| Maple Glen | 12.06.2024 | 21.07.2024 | 02.09.2024 | 29.09.2024 |
| OAC Musca | 12.06.2024 | 21.07.2024 | 02.09.2024 | 29.09.2024 |
| OAC Vision | 12.06.2024 | 21.07.2024 | 28.08.2024 | 11.09.2024 |
| AC Albatros | 12.06.2024 | 21.07.2024 | 02.09.2024 | 11.09.2024 |
| OAC Wallace | 12.06.2024 | 21.07.2024 | 02.09.2024 | 11.09.2024 |
| Naya | 12.06.2024 | 21.07.2024 | 02.09.2024 | 29.09.2024 |
| Saska | 12.06.2024 | 21.07.2024 | 02.09.2024 | 29.09.2024 |
| Narita | 12.06.2024 | 21.07.2024 | 02.09.2024 | 29.09.2024 |
| Ezra | 12.06.2024 | 21.07.2024 | 02.09.2024 | 29.09.2024 |

**Table S 3**.: Estimates and p-values resulting from the linear mixed models.

Shoot biomass is the aboveground biomass including shoots, leaves, pods, seeds etc. The total biomass is the sum of shoot biomass and root biomass. Stover is the shoot biomass minus the seed mass. Significant p-values (<0.05) are highlighted in bold.

|  |  | **Cultivar year of release** | |  | **Treatment**  **(transparent – green)** | |  | **Interaction** | |
| --- | --- | --- | --- | --- | --- | --- | --- | --- | --- |
| Stage of phenological development | Phenotypic parameter | Estimates | P values |  | Estimates | P values |  | Estimates | P values |
| R2 | Plant height | -0.00201 | 0.283 |  | -0.0064 | 0.711 |  | 0.0008 | 0.147 |
| R6 | Plant height | -0.00076 | 0.572 |  | -0.06604 | **0.044** |  | 0.00004 | 0.973 |
| R8 | Plant height | -0.00037 | 0.805 |  | -0.01518 | 0.397 |  | 0.00012 | 0.853 |
| R2 | Root biomass | 0.00059 | 0.606 |  | 0.14917 | **<0.001** |  | 0.00264 | 0.062 |
| R6 | Root biomass | 0.0044 | 0.226 |  | -0.03252 | 0.330 |  | 0.00184 | 0.153 |
| R8 | Root biomass | 0.00863 | **0.017** |  | 0.0883 | **0.005** |  | -0.00321 | **0.006** |
| R2 | Shoot biomass | -0.00197 | **0.036** |  | 0.09436 | **0.010** |  | 0.00155 | 0.248 |
| R6 | Shoot biomass | 0.00359 | 0.058 |  | -0.03255 | 0.061 |  | 0.00085 | 0.187 |
| R8 | Shoot biomass | 0.00293 | 0.059 |  | 0.00603 | 0.647 |  | 0.00048 | 0.340 |
| R2 | Total biomass | -0.00129 | 0.115 |  | 0.11003 | 0.002 |  | 0.00193 | 0.130 |
| R6 | Total biomass | 0.00366 | 0.084 |  | -0.03226 | 0.082 |  | 0.00097 | 0.164 |
| R8 | Total biomass | 0.00351 | **0.046** |  | 0.0125 | 0.337 |  | 0.00011 | 0.822 |
| R2 | Shoot biomass : root biomass | -0.00256 | **0.043** |  | -0.0548 | **0.045** |  | -0.00109 | 0.294 |
| R6 | Shoot biomass : root biomass | -0.00081 | 0.697 |  | -0.00004 | 0.999 |  | -0.00099 | 0.258 |
| R8 | Shoot biomass : root biomass | -0.00569 | **0.016** |  | -0.08227 | **0.005** |  | 0.00369 | **0.001** |
| R2 | Shoot biomass : height | 0.00003 | 0.98543 |  | 0.10077 | **0.00137** |  | 0.00076 | 0.4752 |
| R6 | Shoot biomass : height | 0.00436 | **0.01614** |  | 0.03349 | 0.21943 |  | 0.00081 | 0.42773 |
| R8 | Shoot biomass : height | 0.0033 | 0.08511 |  | 0.02121 | 0.18146 |  | 0.00036 | 0.54608 |
| R8 | Branching | -0.00369 | **0.004** |  | 0.06506 | 0.115 |  | -0.00047 | 0.757 |
| R8 | Seed production | 0.00333 | **0.019** |  | 0.00712 | 0.606 |  | 0.00113 | **0.042** |
| R8 | Seed production : shoot biomass | 0.00039 | 0.37235 |  | 0.00109 | 0.87313 |  | 0.00064 | **0.02095** |
| R8 | Seed production : root biomass | -0.0053 | **0.0378** |  | -.08118 | **0.00897** |  | 0.00433 | **0.00062** |
| R8 | Seed production : total biomass | -0.00018 | 0.76681 |  | -0.00538 | 0.50116 |  | 0.00102 | **0.00296** |
| R8 | Stover | **0.00259** | 0.139 |  | 0.00571 | 0.712 |  | -0.00007 | 0.907 |
